# Spatio-temporal dynamics of root water uptake and identification of soil moisture thresholds for precision irrigation in a Mediterranean yellow-fleshed kiwifruit orchard

**DOI:** 10.3389/fpls.2024.1472093

**Published:** 2024-11-01

**Authors:** Maria Calabritto, Alba N. Mininni, Roberto Di Biase, Angela Pietrafesa, Bartolomeo Dichio

**Affiliations:** Department of European and Mediterranean Cultures, Environment, and Cultural Heritage (DiCEM), University of Basilicata, Matera, Italy

**Keywords:** readily available water (RAW), plant water status, leaf gas exchange, water deficit, precision irrigation management

## Abstract

**Introduction:**

Actinidia is highly susceptible to water stress, both excess and shortage, and is therefore a model fruit crop for irrigation management, requiring precise water application. The present study was carried out in a mature kiwifruit orchard in southern Italy to investigate the physiology of a yellow-fleshed kiwifruit cultivar under non-limiting soil water conditions and in response to a progressive decrease in soil water content in a Mediterranean environment, with the aim of defining soil moisture thresholds to guide irrigation management.

**Methods:**

The progressive lowering in soil moisture was monitored using multi-profile probes, taking into account a 60 cm layer. Plant water status and physiological parameters were measured throughout the experiment and were significantly correlated with soil water status, suggesting that the level of soil water deficit affects plant physiological performance.

**Results:**

Reference minimum values of stem water potential reached during the day under non-limiting soil water conditions ranged from -0.4 to -0.7 MPa, with a value of -0.8 MPa identifying the threshold below which stomatal conductance began to decrease significantly. Soil moisture thresholds were defined according to the spatio-temporal dynamics of available water (AW) reduction, which decreased by approx. 10% and 1% before the onset of water stress and 16% and 2% at the onset of water stress, considered in the 0-30 cm and 30-60 cm soil layers, respectively, compared to the AW content of the whole soil profile.

**Discussion:**

Results confirmed that root uptake was mainly concentrated in the first 30 cm of soil depth, which should be properly managed by irrigation, as reduced soil water availability could easily lead to plant water stress. An integrated approach, combining plant measurements and soil water content monitoring, together with an assessment of root water uptake dynamics, is essential to identify soil water thresholds and develop precision irrigation, especially for high water-demanding crops and environments.

## 1 Introduction

Mediterranean environments are characterized by increasingly high temperatures, light intensities and vapor pressure deficit, combined with low precipitations, especially during the summer period. This affects the physiological behavior of plants and requires a consistent use of water for irrigation, the availability of which is severely compromised (Xiloyannis et al., 2012). The variability of climatic parameters induced by climate change affects several aspects of plants, altering phenological stages and modifying physiological processes, yield and qualitative traits (Medda et al., 2022).

In recent years, economic and market pressures have led to major investments in kiwifruit production, with more than 26,000 hectares under cultivation in Italy. Kiwifruit, which originated in China, has grown in habitats characterized by relatively high humidity, abundant rainfall and a moderate light intensity (Ferguson, 1984). Despite the climatic conditions in which kiwifruit grows, the commercial importance of this crop has encouraged its cultivation and spread in the semi-arid Mediterranean area. The most commercially important and widespread kiwifruit belongs to the genus Actinidia, in particular the green-fleshed variety (*A. chinensis* var. *deliciosa*), which prefers cold climates and higher altitudes (from western China), is gradually being replaced by the yellow-fleshed variety (*A. chinensis* var. *chinensis*), which is better adapted to warm climates (from eastern or southern China) (Ferguson, 2013; Bardi, 2020).

Kiwifruit has evolved large leaves (wide transpiring surface) and conducting xylem vessels as an adaptation to environments with high soil water availability (Dichio et al., 2013), showing a very low resistance to water movement within the vine and a high transpiration rate (McAneney and Judd, 1983; Chartzoulakis et al., 1993). Numerous tiny fibrous (feeder) roots developing from thicker structural and secondary roots constitute the kiwifruit root system, with high root density and intense development in the upper soil layers, which decrease with increasing soil depth (Gandar and Hughes, 1988).

Due to its anatomical features, kiwifruit is widely recognized as a water-consuming plant with high water requirements (Chartzoulakis et al., 1993; Torres-Ruiz et al., 2016), but at the same time extremely sensitive to water stress, either as water excess (Savian et al., 2020; Sofo et al., 2024) or shortage (Montanaro et al., 2007; Dichio et al., 2013). Water requirement is influenced by environmental and soil characteristics as well as by plant morphology and physiology (Holzapfel et al., 2000). Optimal water application levels in kiwifruit orchards allow for normal transpiration, photosynthetic activity and overall physiological functioning of the plant. Due to the high sensitivity of kiwifruit to different levels of water application, special attention should be paid to irrigation management to ensure water distribution in the right amount, at the right time and in the right place in order to improve the physiological performance of kiwifruit vines and to develop new and more efficient precision irrigation strategies for this crop. On the one hand, optimized irrigation management and proper scheduling of the amount and timing of water supply can be achieved by evaluating plant water status (Virnodkar et al., 2020). Plant water status represents a sensitive indicator of water stress, measuring the plant response to the combined effects of soil moisture availability, environmental evaporative demand, hydraulic resistances and root uptake capacity (Ihuoma and Madramootoo, 2017). Predawn and midday water potentials have been the most commonly used indicators of plant water status and stress for irrigation scheduling in fruit orchards (Naor and Cohen, 2003). On the other hand, soil water content monitoring has recently become a key factor in irrigation optimization, as it provides important information for better estimation of crop water demands (Karandish and Šimůnek, 2016), and sensor-based soil moisture measurements seem to effectively improve irrigation water application (El-Naggar et al., 2020). Changes in soil water content in the root zone, which reflect the combined history of root water uptake and recent wetting by irrigation, can be effectively monitored using soil moisture probes (Clothier and Green, 1994). Plants take up soil water in the root zone, gradually depleting the available water to meet the evapotranspiration needs (Ihuoma and Madramootoo, 2017), which are considered to be unreduced as long as the readily available water (RAW) is used by plants (Ferreira et al., 2017). Therefore, efficient irrigation management also depends on knowing the spatial and temporal distribution of root water uptake, as well as predicting changes in soil water status in the root zone after irrigation (Koumanov et al., 2006), which can be easily detected by soil moisture probes. Consideration of the soil volume explored by the root system is a key issue in defining the most effective irrigation strategy (McAneney and Judd, 1983), as the distribution of fine roots affects the potential water uptake activity, so it might be expected that the pattern of uptake would be similar to the distribution of fine roots under non-limiting water conditions (Green and Clothier, 1995). The hyperactivity of the near-surface roots is revealed by the rapidly changing trends in soil water content monitored in the first layers, approximately 0-20 cm (Green et al., 2006). An initial good correspondence between water uptake and root density was found under well-watered conditions in the rootzone, while as water stress developed, the deeper layers, where water was more freely available, began to contribute an increasing fraction of total water uptake, despite their lower root density (Green and Clothier, 1995). While there are some investigations on kiwifruit root morphology and distribution (Greaves, 1985; Gandar and Hughes, 1988; d'Andria et al., 1991), more information is needed to improve knowledge of the spatial and temporal distribution of root water uptake, especially under different localized irrigation application strategies. Soil water content thresholds, defined as the lower or upper limits of soil water content for irrigation, are important to provide the most suitable soil water environment for optimal kiwi performance (He et al., 2023) and should take into account water uptake dynamics.

Soil water observations combined with plant measurements and responses are essential for implementing suitable and more accurate irrigation scheduling methods (Intrigliolo and Castel, 2004), requiring early detection of water stress (Green et al., 2015).

Conflicting opinions have been reported on stomata control and the ability of kiwifruit to reduce water loss under water restriction. Stomatal regulation, which is involved in the control of water loss and plant water potential during water stress, differs among species but also between cultivars due to adaptive responses that have evolved to limit severe drops in potentials (Álvarez-Maldini et al., 2022), leading to the distinction between isohydric and anisohydric behavior. Gucci et al. (1996) found that stomata were highly sensitive to changes in soil water content, effectively regulating transpiration processes in Hayward kiwifruit. In contrast, other research has shown that leaves were unable to regulate stomatal conductance with increasing soil water deficit, continuing to lose water (Judd et al., 1989). It is still unclear whether and how drought affects the physiological responses and stomatal behavior of kiwifruit at the field scale. As soil water potential decreases, leaf stomata influence gas exchange dynamics and the feedback mechanism between plant water potential and stomatal conductance is crop-specific and varies among species (Jamshidi et al., 2020). While most studies on water relations and plant physiological processes have been carried out on the well-known green cultivar 'Hayward', less is known about the yellow cultivars and associated crop management protocols (Mills et al., 2009). Mills et al. (2009) reported better stomatal control under water stress in yellow kiwifruit compared to the green cultivar, while Black et al. (2012) found reductions in ψ_leaf_ without significant reductions in stomatal conductance, noting that the observed reductions in ψ_leaf_ were insufficient to induce a stomatal response. Previous studies have found that the anisohydric or isohydric behavior of cultivars also depends on soil hydraulic conductivity and water content (Tramontini et al., 2014), suggesting a great influence of the environment and a plant-environment interaction rather than an intrinsic property of the plant behind the discriminated behavior (Hochberg et al., 2018). The combination of soil water status, which shows a direct relationship with the amount of irrigation water applied (Holzapfel et al., 2000), with plant physiological responses could effectively help to modulate and manage the water content in the soil profile to ensure improved plant functioning. Therefore, the main objectives of this study were: (1) to further characterize the physiology of yellow-fleshed kiwifruit under non-limiting soil water availability in a typical Mediterranean environment; (2) to evaluate the effects of progressive soil water depletion on the behavior of yellow-fleshed kiwifruit in order to identify RAW thresholds for precision irrigation that ensure optimal plant physiological performance, and levels below which plant water status and physiological parameters are significantly affected; (3) to investigate the spatial pattern of root water uptake, considering the dynamics of available water (AW) depletion, to develop and match precision irrigation strategies to the pattern of plant water extraction from the soil profile.

## 2 Materials and methods

### 2.1 Experimental site and plant material

The experimental trial was carried out during the 2022 growing season over a 4-week period from 23 August to 19 September (DOE=days of the experiment) in a 9-year-old mature commercial kiwifruit (*Actinidia chinensis* var. *chinensis*, cv. Zesy002) orchard in Metaponto, southern Italy (latitude 40°24'N, longitude 16°46'E). The yellow-fleshed cultivar was grafted onto D1 rootstock (*A. chinensis* var. *deliciosa*), cultivated at 5 m x 2 m spacing and trained as a pergola system. The soil profile consisted of a uniform sandy clay loam texture (58% sand, 14% silt and 28% clay).

Irrigation was provided by micro-sprinklers, using a single emitter per vine spaced 1 m from the trunk, at a flow rate of 40 L h^-1^ and with a wetted radius of 0.9 m. Soil volume wetted by irrigation, expressed as total volume per hectare (m^3^ ha^-1^), was assumed as a cylinder and calculated considering the sprinkler wetting radius, the depth of the wetting pattern (0.6 m) and the number of emitters per hectare. All vines were grown under optimal water availability conditions during the season until the beginning of the experimental trial. Daily vine water requirement was estimated through the compilation of the water balance, taking into account environmental data and crop coefficient (K_c_) reported in the FAO Irrigation and Drainage paper Nr. 66 (Steduto et al., 2012). The scheduled irrigation volume was adjusted throughout the season through the soil water balance, operating feedback adjustment mechanisms based on the monitoring of the water content in the soil volume wetted by irrigation by soil moisture probes, in order to maintain the soil moisture level close to or slightly below the field capacity (FC) (Vera et al., 2019; Mininni et al., 2022). Thus, irrigation drainage in this study was considered negligible. The experimental irrigation-managed plot, consisting of 63 vines, was equipped with digital flow meters and an automatic irrigation controller for remote control of water supply ("Irrifarm" system, Pan. Agri s.r.l., Basilicata, South Italy). Daily irrigation volumes ranged from 30 to 60 m^3^ ha^-1^ during the season.

From 23 August on, 12 vines within the experimental plot and distributed along a row, underwent restricted irrigation (WS) with irrigation volumes reduced by 30% and ceased, adjusted taking into account rainfall contribution, to allow the soil to achieve a progressive available water content (AW) depletion. The soil about the control vines (C) received optimal irrigation, ensuring a soil water content level close to or slightly below the FC, so that it remained well-watered throughout the experiment. Half of the water-stressed vines were re-irrigated after 15 days (R_I_) following the irrigation strategy kept for the control vines, restoring the available water content in the soil profile, while the remaining half of the vines continued to be subjected to restricted irrigation for other 5 days (WS), subsequently entering the recovery phase (R_II_).

### 2.2 Meteorological data and soil water status monitoring

A meteorological station was installed nearby the experimental orchard block and mounted above the kiwifruit canopy, outside the hail net protection. The sensors recorded hourly averages of wind speed, air temperature, relative humidity, evapotranspiration and daily rainfall. Hourly air vapor pressure deficit (VPD) was computed using air temperature and relative humidity.

Frequency Domain Reflectometry (FDR) 90 cm multi-profile soil moisture probes (Drill & Drop, Sentek Sensor Technologies Stepney, Australia) were used to continuously monitor the volumetric soil water content in 10 cm increments throughout the soil vertical profile, allowing the remote detection of instantaneous oscillations and trends in each soil layer. A soil layer of 0-60 cm was considered useful for irrigation scheduling in kiwifruit (Xiloyannis et al., 2023). Sensors were installed on the row at a distance of 0.5 m from the vine stem and approximately in the center of the volume of wet soil. Measured values of soil moisture were stored in 15-min-intervals using a data logger (Model CR1000, Campbell Scientific, USA), hourly means were considered for analyses.

### 2.3 AW depletion and spatial pattern of root-water uptake

The soil physico-hydrological properties were evaluated through the soil-water retention curve obtained with the Richards' pressure plates apparatus (Richards, 1948) to define the soil-water relation and, in particular, the quantity of water held at different values ​​of soil matric potential, and therefore the FC, the wilting point (WP) and the AW in the whole profile (0-60 cm) (**Table 1**).

**Table 1:** Soil physico-hydrological characteristics and available water contents in the soil profile (0-60 cm) and in different soil layers (0-20, 20-40, 40-60 cm).

Soil properties	Value
Soil volume wetted by irrigation (m^3^ ha^-1^)	1,526
Bulk density (kg dm^-3^)	1.39
Field Capacity (cm^3^ cm^-3^)	0.39
Wilting Point (cm^3^ cm^-3^)	0.18
Available Water (m^3^ ha^-1^) (0-60cm)	320.30
Available Water (m^3^ ha^-1^) (0-20 cm)	106.77
Available Water (m^3^ ha^-1^) (20-40 cm)	106.77
Available Water (m^3^ ha^-1^) (40-60 cm)	106.77

The available water (AW, m^3^ ha^-1^) that can be contained in each layer, and thus in the whole 0-60 cm soil profile considered in the present study, was calculated taking into account soil water retention characteristics derived from the soil-water retention curve (FC and WP values, **Table 1**) and soil volume of each 10 cm layer wetted by irrigation calculated on a per hectare basis, which is influenced by the irrigation system and the wetted radius. The volumetric soil water content values recorded by the soil moisture probes in 10 cm increments were used to estimate the actual available water (AWact, m^3^ ha^-1^) contained in each soil layer and in the whole 0-60 cm profile on a per hectare basis. The depletion of AW (AWdepletion, %) was calculated using the following equation:


$$AWdepletion\;\left(\%\right)\;=\frac{AW\;-\;AWact}{AW}*100\;$$

where AW is the potential available water contained in each soil layer (10 cm increments) or in the whole profile (0-60 cm) and AWact is the actual value of the available water calculated for each day of the experiment.

FDR technologies, which allowed for routine measurements and detection of temporal changes in the soil water content over time, were used to deduce the spatial pattern of water uptake by the roots in the soil profile. In Mediterranean environments, the volume of soil not wetted by irrigation tends to dry out during the warmer months, so it is not affected by root water uptake (Green and Clothier, 1995). Drainage of water in the deeper layer was nullified by the precise irrigation management, which was based on the continuous monitoring of soil water content and the distribution of daily irrigation volumes in two/three events per day, thus it was considered negligible in the present study. Soil evaporation plus understorey transpiration were minimized due to localized micro-irrigation, high vine cover and absence of cover crops, making irrigation requirements almost dominated by vine transpiration (Goodwin et al., 2004; Goodwin et al., 2015). Therefore, they were assumed to be not relevant for irrigation management purposes in the present study.

### 2.4 Plant water status and leaf gas exchange

Diurnal courses of physiological stress indicators were measured to investigate plant-water relations by analyzing their trends influenced by changing soil water status conditions, both for stressed and well-watered vines, and considering the peculiar physiological and anatomical traits of kiwifruit vines. Physiological measurements were performed during the increasing soil water deficit conditions (WS vines) and the subsequent recovery phases (R_I_ and R_II_ vines) compared to C vines.

Plant water status and stress level were monitored through the stem water potential (ψ_stem,_ MPa) on six fully developed, expanded and not exposed to sunlight leaves chosen from the middle part of the canopy of three plants per treatment. The selected leaves were wrapped in a tin foil for about half an hour and then excised for immediate measurement with a Scholander pressure chamber (Model 1000, PMS Instrument Company, Corvallis, OR, USA) pressurized with nitrogen according to the procedure recommended by Begg and Turner (1970). The ψ_stem_ daily trends were monitored on seven days of the experiment at two-hourly intervals throughout the day, from 5:30 (pre-dawn values) to 16:30 (afternoon values) hours (European daylight savings time, DST), in order to understand the synchronization of ψ_stem_ diurnal pattern with soil volumetric water content and VPD environmental conditions. Stomatal conductance (gsw, mol m^-2^ s^-1^), transpiration (E, mmol m^-2^ s^-1^) and leaf temperatures (T_leaf_, °C) were similarly monitored during the same days with a portable handheld LI-600 porometer system integrated with a fluorometer (LI-COR Biosciences, Lincoln, USA). Values obtained were averages of nine measurements taken on mature and sun-exposed leaves, near the top of the canopy, with no visual symptoms of biotic or abiotic stress, from three vines per treatment. During the other days (five) of trial observation, measurements of ψ_stem_, gsw, E and T_leaf_ were conducted at midday (between 12:30 and 14:30 hours) on sunny days. Leaf to air temperature differences (ΔT (°C) = T_leaf_ - T_air_) were calculated. Measurements of ψ_stem_, gsw and E were carried out at 1, 2, 5, 7, 12 and 2, 7 days after re-watering for the first and second recovery phases (R_I_ and R_II_) compared to C. Measurements of photosynthesis (A, µmol m^-2^ s^-1^) were carried out on six fully expanded, developed and sun-exposed leaves from three vines during the drought (diurnal trends for four days) and re-watering (midday values for two days) cycles with an open gas exchange system unit (ADC LCA-4, Analytical Development Co., Hoddesdon, UK).

### 2.5 Statistical analysis

All statistical analyses were performed using RStudio statistical software (4.2.2 version, Posit Software, PBC, Boston, MA) and results were plotted with SigmaPlot 15.0 (Systat Software Inc., San Jose, CA, USA). Data were reported as mean value and standard error of the mean (± SE). The data were subjected to an analysis of variance (one-way ANOVA) to examine the differences between irrigation treatments at each sampling time (p value<0.05 was considered significant) after testing for normality distribution of residuals (Shapiro-Wilk test) and homogeneity of variances (Levene's test). Dunnett's test was used for mean comparison of WS and R_I_ against the control. To investigate the relationships between physiological indicators (ψ_stem_, gsw, A) and soil water status (SWC), polynomial regression models were fitted to averaged data from each irrigation treatment.

## 3 Results

### 3.1 Environmental conditions and progressive soil water depletion

**Figure 1:**
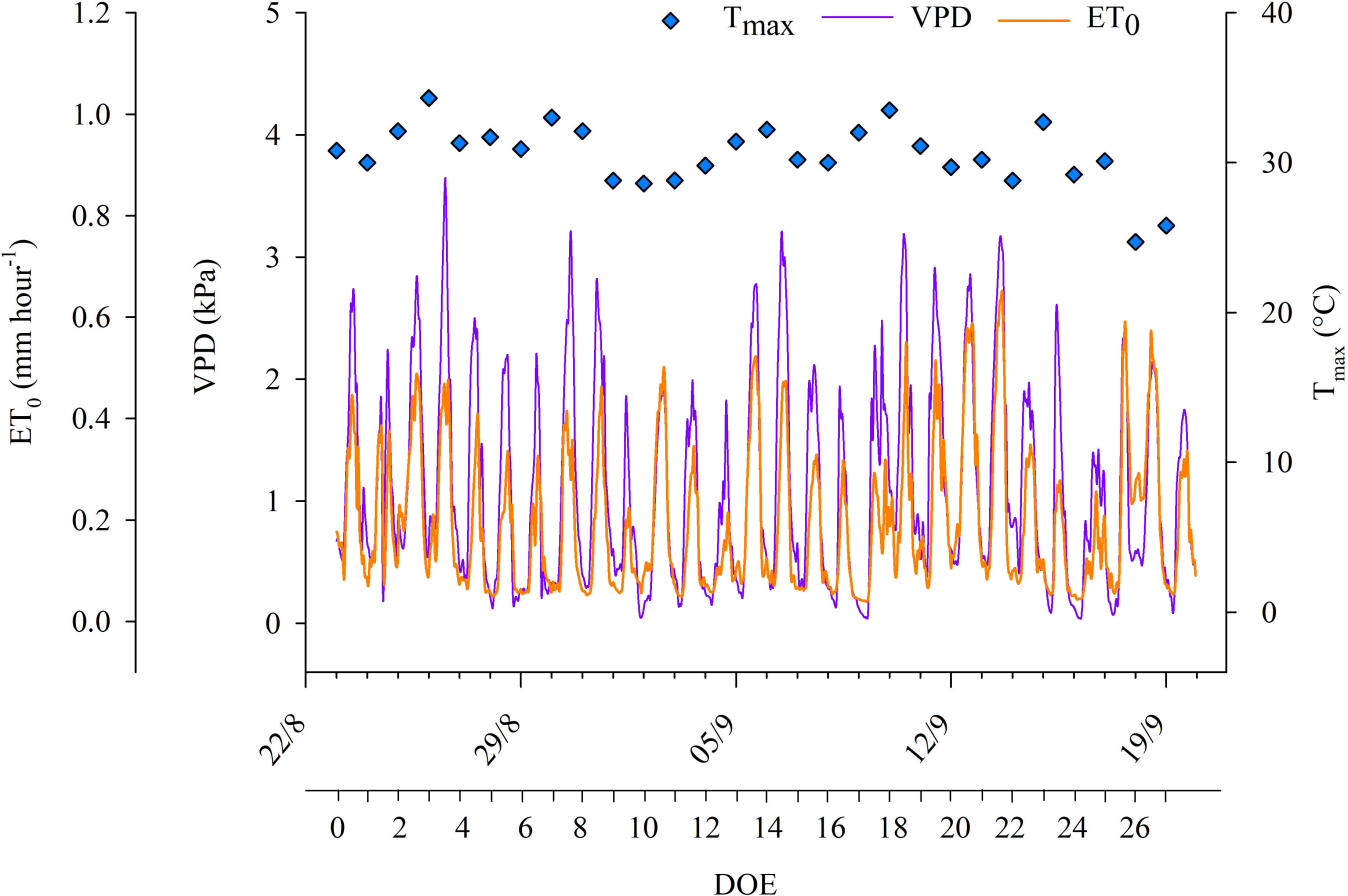
Hourly reference evapotranspiration (ET_0_), hourly vapor pressure deficit (VPD) and daily maximum air temperatures (T_max_) during the days of the experiment (DOE) carried out in the 2022 season.

The experimental trial coincided with a period characterized by a quite high evaporative demand. The weather was generally sunny and dry with the exception of a few days when rainfall occurred. 19 days out of the 28 days had maximum air temperatures that exceeded 30°C (**Figure 1**), while the minimum air temperatures varied between 9 and 24°C during the experimental trial. VPD was quite variable throughout the experimental days, reaching maximum hourly values equal to 3.65 kPa at DOE 3. Daily ET_0_ ranged between 3.5 and 6.0 mm. Daily net solar radiation reached a maximum value of 24.2 MJ m^-2^ (DOE 3) and ranged between 16.2 and 24.2 MJ m^-2^ during the experimental trial. Irradiance, air temperature and humidity were typical of Mediterranean late summer conditions.

**Figure 2:**
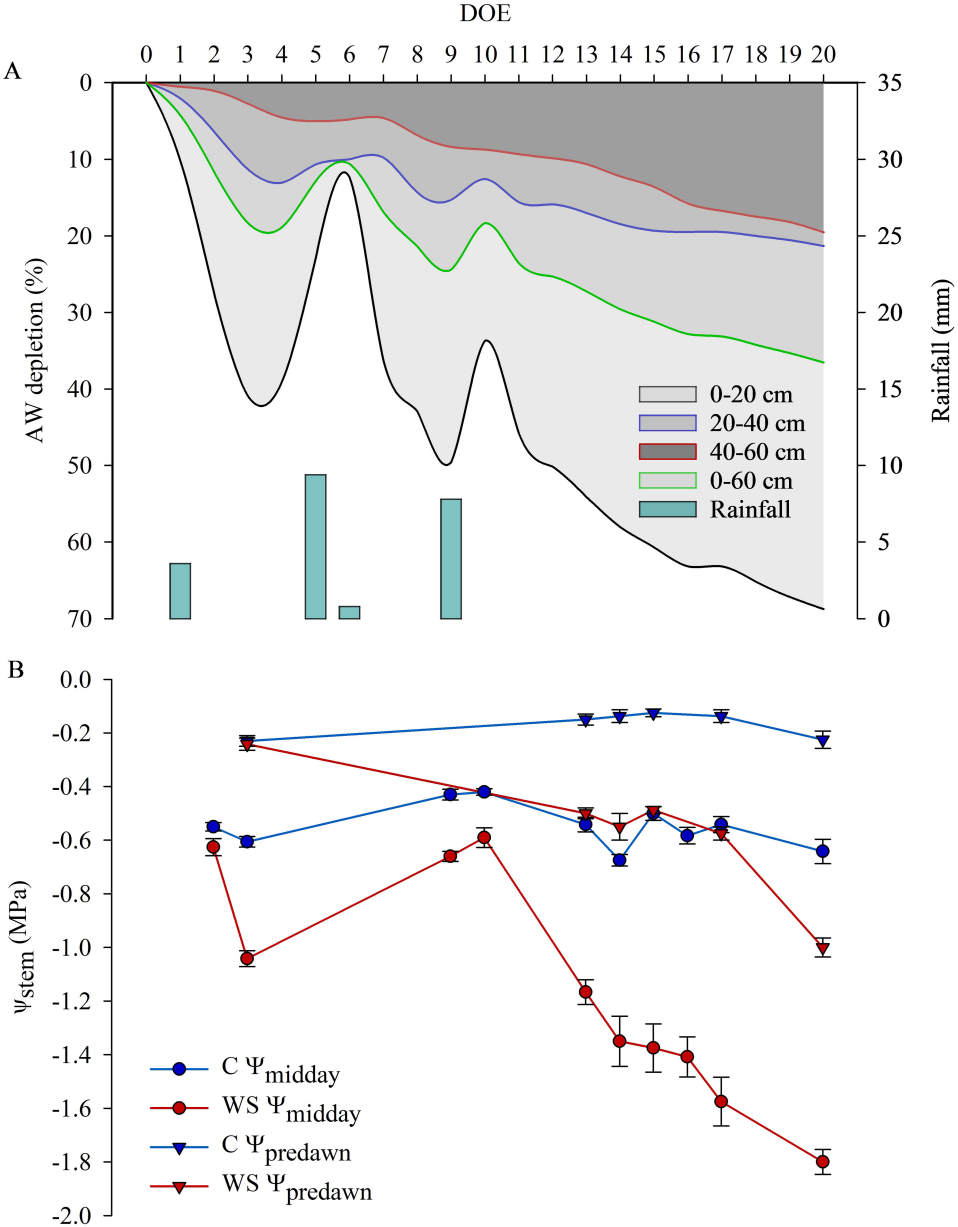
Daily rainfall and available water (AW) depletion averaged in the 0-20, 20-40, 40-60 cm layers and in the 0-60 cm soil profile **(A)**, daily midday and pre-dawn stem water potential measured in control (C) and water stressed (WS) vines **(B)** during the days of the experiment (DOE) of restricted irrigation.

Soil water content gradually decreased in the field area subjected to water restriction and trends of AW depletion in different soil layers and in the whole soil profile are shown in **Figure 2A**. In particular, AW was reduced by approx. 61%, 19% and 14% in the 0-20, 20-40 and 40-60cm soil layers, respectively, at the end of the first phase of restricted irrigation (DOE 15) before the stress was relieved by re-watering vines entering the R_I_ recovery phase. An AW depletion of approx. 69%, 21% and 19% computed in the 0-20, 20-40 and 40-60 cm soil layers, respectively, was achieved at the maximum level of stress (DOE 20) before the stress was relieved by re-watering vines entering the R_II_ recovery phase (**Figure 2A**). In C vines soil water content was on average maintained close to or slightly below the FC. As water stress increased and in response to progressive soil water depletion, ψ_stem_ of WS vines became more negative, reaching pre-dawn values (ψ_predawn_) of -0.5 and -1.0 MPa, and midday values (ψ_midday_) of -1.4 and -1.8 MPa, respectively at the end of the first and the second phase of restricted irrigation, when about 31% and 36.5% of AW in the whole soil profile (0-60 cm) was depleted (**Figures 2A, B**). C vines maintained ψ_stem_ values indicative of an optimal plant water status, ranging from -0.1/-0.2 MPa at pre-dawn to -0.4/-0.7 MPa at midday throughout the days of the experiment of restricted irrigation (**Figure 2B**).

### 3.2 Plant water status and leaf gas exchange

**Figure 3:**
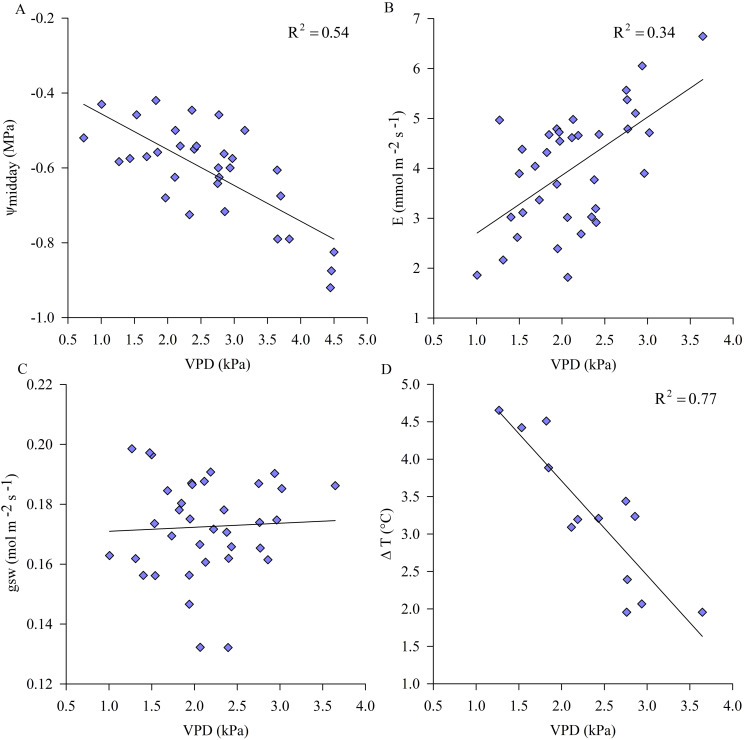
Variations of midday stem water potential (ψ_midday_) **(A)**, transpiration (E). **(B)**, stomatal conductance (gsw) **(C)** and midday leaf to air temperature differences (ΔT) **(D)** to air vapor pressure deficit (VPD) under non-limiting soil water availability (well-irrigated kiwifruit vines). Symbols are means of measured data (ψ_stem_, n=6; E, gsw and T_leaf_, n=9).

Under non-limiting soil water conditions, ψ_stem_ and ΔT observations correlated with midday VPD more tightly than E, whereas the relation between gsw and VPD was not significant (**Figure 3**). Relationships between physiological indicators and air VPD indicated that, under non-limiting soil water conditions, vines did not implement any stomatal regulation with the daily increase in environmental evaporative demand (i.e. VPD) preferring to transpire, losing water derived from root uptake, and slightly lowered the plant water potentials.

**Figure 4:**
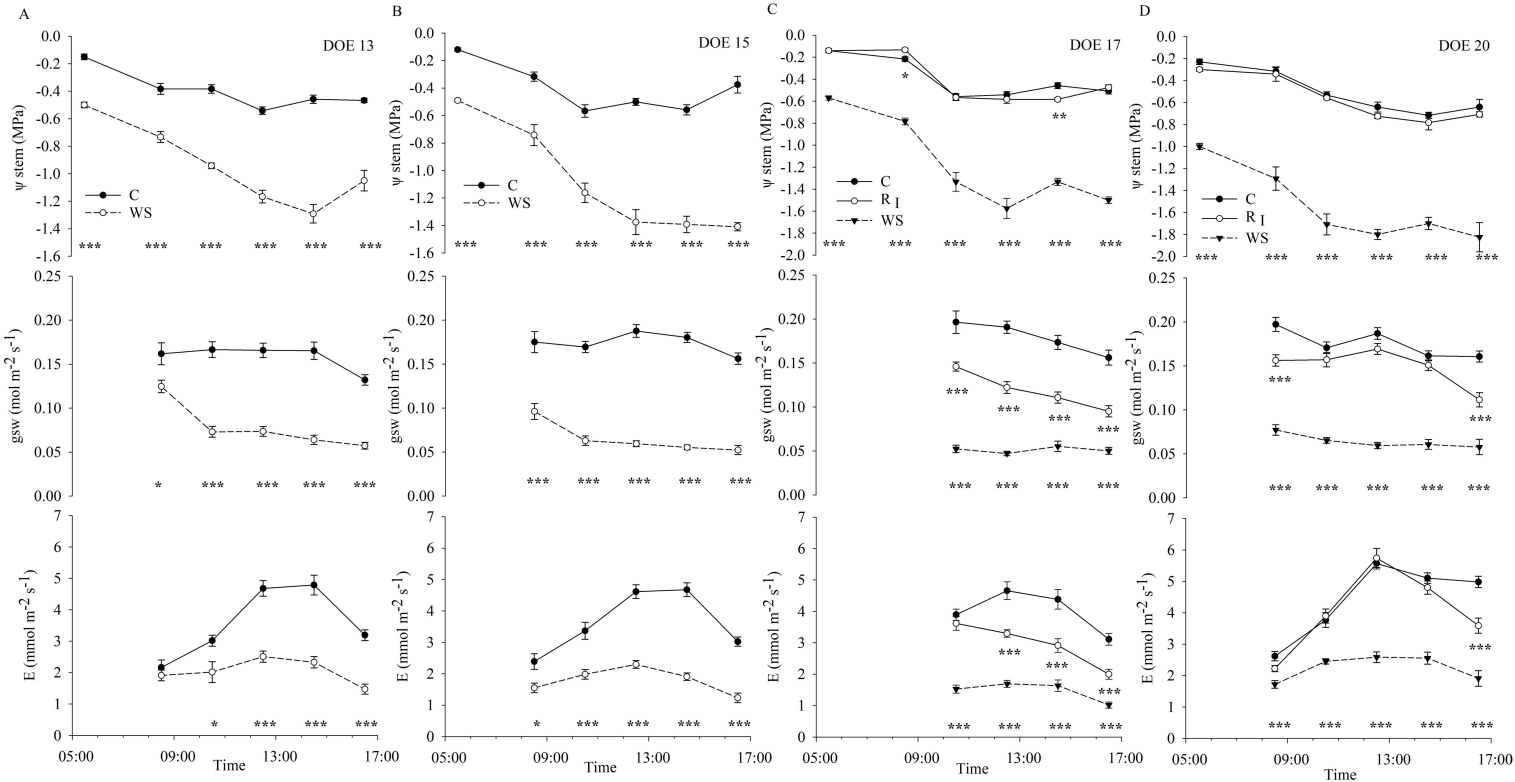
Daily trends of stem water potential (ψ_stem_), stomatal conductance (gsw) and transpiration (E) measured in the first phase of restricted irrigation (DOE 13 **(A)** and DOE 15 **(B)**; C= Control and WS= Water-Stressed) and in the second phase of restricted irrigation and first recovery (DOE 17 **(C)** and DOE 20 **(D)**; C= Control, R_I_= Recovery and WS= Water-Stressed). Each point is the mean of measured data ± SE (ψ_stem_, n=6; gsw and E, n=9). Asterisks denote significant differences in ψ_stem_, gsw and E, respectively, between R_I_ and WS vines compared with C according to a One-Way ANOVA test (*p< 0.05*).

ψ_stem_ of C vines showed daily trends reflecting daily VPD conditions, reaching minimum values during the hottest hours (e.g., around midday), but remaining in an optimal range when soil water availability was not limiting (**Figure 4**), allowing the plant's physiological activities not to be compromised. Vines under limited soil water availability conditions showed a fast drop in ψ_stem_ at midday and a gradual incapacity to recover plant water status in the afternoon hours, as highlighted on DOE 15 (**Figure 4A**), affecting the ability to recover during the nighttime hours, as shown by the progressive lowering of the ψ_predawn_ values (**Figures 2B**, **4**). Stomatal conductance remained relatively constant between 0.16 and 0.20 mol m^-2^ s^-1^, throughout the day and between different days in C vines (**Figure 4**), supporting the results from **Figure 3**. Stomatal conductance of WS vines was higher early in the morning, especially during the first phase of restricted irrigation (**Figures 4A, B**) when ψ_stem_ was not yet the seriously limiting factor, then gradually decreased and remained low throughout the day, reduced by approx. 55-75% compared with C vines during the days of experiment of significant stress (**Figures 4C, D**), reaching values relatively constant between 0.05 and 0.08 mol m^-2^ s^-1^, probably due to the implementation of an adaptive strategy involving stomatal control as water is depleted in the root zone. However, stomatal closure, occurring in the WS vines after the early morning hours, did not prevent ψ_stem_ from a further drop in the afternoon hours (**Figures 4A, B**). Conversely to gsw, E measured in kiwifruit vines under non-limiting soil water conditions was affected by VPD, increasing when environmental conditions became more water-demanding (**Figure 3B**). Transpiration rate in C vines was low in the early morning due to lower light intensity and air VPD. With increasing light intensity and temperature, E rose reaching higher values at midday and then gradually decreased (**Figure 4**). In WS vines, E generally remained at very low values throughout the day, reflecting the same daily trend as in C vines. In particular, E of WS vines measured at early morning were similar to those of C vines in the first phase of restricted irrigation, also aided by low environmental demand, while they significantly differed during hours of higher evaporative demand (**Figures 4A, B**). Instead, E of WS vines differed since early morning from that of C vines in the second phase of restricted irrigation (**Figures 4C, D**), achieving a reduction of approx. 50-65% compared with control, almost constant over the days.

**Figure 5:**
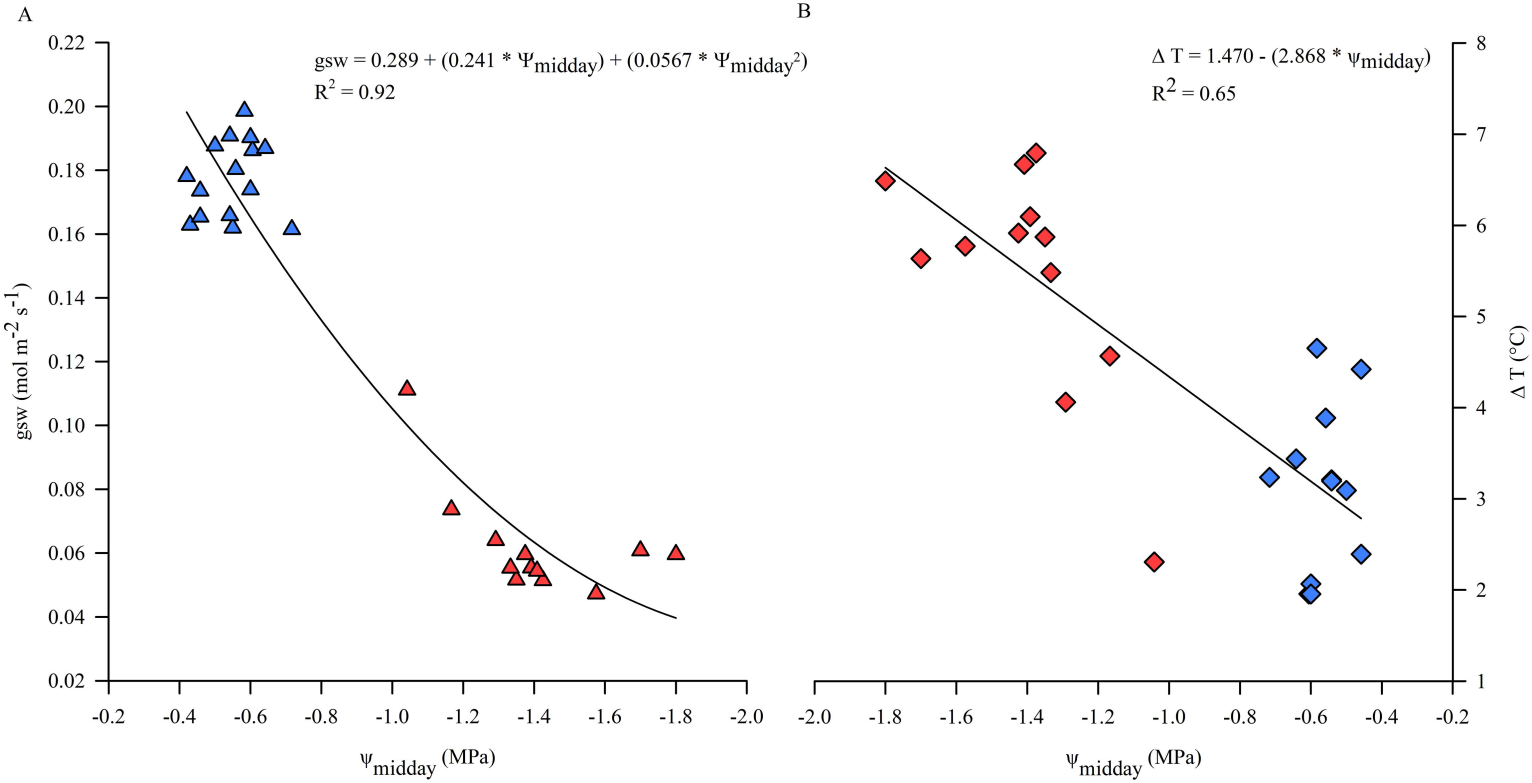
Correlation between midday stem water potential (ψ_midday_) and stomatal conductance (gsw) **(A)** and leaf to air temperature differences (ΔT) **(B)** during the experimental trial. Symbols are means of measured data, blue for optimal irrigation level and soil water availability; red for restricted irrigation and progressive soil water content lowering. Line corresponds to modeled data.

Leaf temperature of sun exposed leaves of kiwifruit vines under non-limiting soil water conditions, monitored during the midday hours (12:30-14:30 hours), was higher than air temperature in the range of approx. 2-4.5°C, depending on daily VPD conditions. In particular, leaf to air temperature differences (ΔT) decreased with increasing VPD and E, due to the potential cooling effect of transpiration (**Figure 3D**). Otherwise, in WS vines T_leaf_ were nearly 4°C higher than in C vines on some days of experiment (data not shown) and ΔT, on average equal to approx. 6°C, increased due to the reduced transpiration rate and consequent loss of thermoregulatory capacity under water stress conditions (**Figure 5B**). Reference values of ψ_midday_, allowing maximum stomatal activity and opening, ranged from -0.4 down to -0.7 MPa, under non-limiting soil water conditions (**Figure 5A**). Results showed that under a threshold value of ψ_midday_, generally corresponding to the minimum reached during the day, of approx. -0.8 MPa, gsw began to decrease significantly while ΔT increased (**Figure 5**), indicating a change in plant behavior when subjected to water stress conditions.

Vines were then subjected to a recovery phase (R_I_ and R_II_) from a different level of water stress, indicated by ψ_midday_ and ψ_pre-dawn_ values of approx. -1.4 and -0.5 MPa reached at the end of the first phase of restricted irrigation, and -1.8 and -1.0 MPa reached at the end of the second phase of restricted irrigation, respectively. Recovery dynamics of physiological indicators are shown in **Table 2**. Upon re-watering, recovery of ψ_stem_ was rapid both in the first and in the second recovery (R_I_ and R_II_), restoring values equal to those of C vines in the immediate 24 hours, regardless of the water stress level achieved. However, gsw of WS vines showed a delay in recovery, reaching 86% and 51% of the value of C vines one week after re-watering from the first and the second phase of restricted irrigation, respectively, showing a different recovery trend depending on the stress level achieved. Similarly, E of WS vines recovered slowly, but not as slowly as gsw, showing no differences with C vines and reaching 76% of the value of C vines 5 days and one week after re-watering from the first and the second phase of restricted irrigation, respectively (**Table 2**).

**Table 2:** Midday stem water potential (ψ_midday_), stomatal conductance (gsw) and transpiration (E) measured in the first (R_I_) and in the second recovery (R_II_) at several days after re-watering from a condition of pre-dawn stem water potential (ψ_pre-dawn_) values of approx. -0.5 and -1.0 MPa, respectively.

Recovery days	Treatment	gsw (mol m^-2^ s^-1^)	E (mmol m^-2^ s^-1^)	ψmidday (MPa)
	C	0.199 ± 0.01	4.97 ± 0.20	-0.58 ± 0.03
1	R _I_	0.107 ± 0.01	2.69 ± 0.22	-0.56 ± 0.02
	Significance	***	***	ns
	C	0.191 ± 0.01	4.66 ± 0.28	-0.54 ± 0.03
2	R _I_	0.122 ± 0.01	3.30 ± 0.12	-0.58 ± 0.04
	Significance	***	***	ns
	C	0.187 ± 0.01	5.57 ± 0.17	-0.64 ± 0.05
5	R _I_	0.169 ± 0.01	5.74 ± 0.30	-0.73 ± 0.02
	Significance	ns	ns	ns
	C	0.187 ± 0.01	4.72 ± 0.17	-0.68 ± 0.02
7	R _I_	0.161 ± 0.01	4.26 ± 0.23	-0.66 ± 0.04
2	R _II_	0.065	1.99 ± 0.16	-0.75 ± 0.05
	Significance C vs. R _I_	**	ns	ns
	Significance C vs. R _II_	***	***	ns
	C	0.185 ± 0.01	4.04 ± 0.22	-0.57 ± 0.03
12	R _I_	0.150 ± 0.01	3.33 ± 0.20	-0.64 ± 0.02
7	R _II_	0.095 ± 0.01	2.52 ± 0.18	-0.80 ± 0.05
	Significance C vs. R _I_	**	*	ns
	Significance C vs. R _II_	***	***	**

Asterisks denote significant differences in ψ_midday_, gsw and E, respectively, between R_I_ and R_II_ vines compared with control vines (C) according to a One-Way ANOVA test (*p< 0.05*).

**Figure 6:**
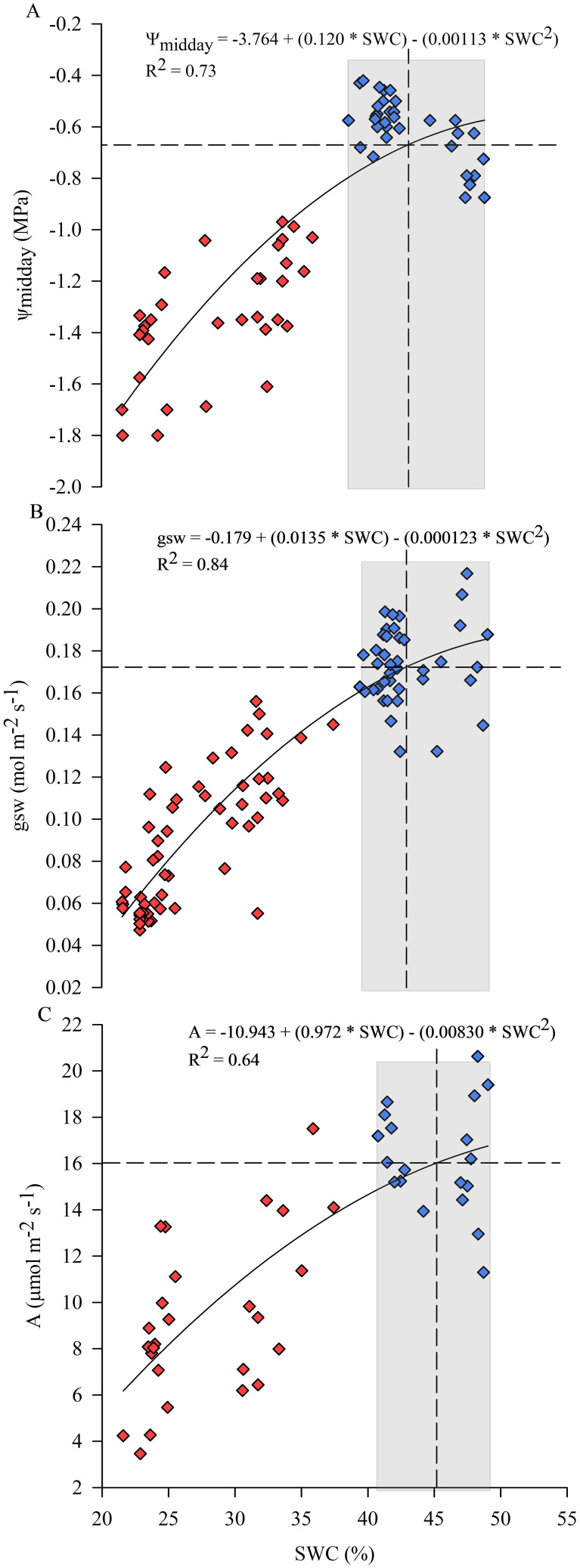
Responses of midday stem water potential (ψ_midday_) **(A)**, stomatal conductance (gsw) **(B)** and photosynthesis (A) **(C)** to soil water content (SWC) considered in the 0-40 cm soil layer during the experimental trial. Symbols are means of measured data, blue for optimal irrigation level and soil water availability, red for restricted irrigation and progressive soil water content lowering. Lines correspond to modeled data. (Some additional measurements taken externally to the experimental period were included in the analysis).

The response of physiological indicators to SWC during the days of experiment is shown in **Figure 6**. In particular, ψ_midday_, gsw and photosynthesis (A) showed a positive correlation with SWC recorded by soil moisture probes in the 0-40 cm soil layer, indicating that a progressive decrease in SWC negatively affected plant behavior (**Figure 6**). Relationships were carried out considering the 0-40 cm soil layer that was mainly affected by root water uptake dynamics as shown in **Figures 2A**, **8**. SWC was maintained approximately in the range between 40% and 50% for C vines in the 0-40 cm soil layer. Stomata were wide open, allowing maximum transpiration rates when SWC was maintained at optimal levels (**Figure 6B**). ψ_midday_, gsw and A declined by 50% once SWC dropped to about 27%, 26% and 24%, respectively. At much lower SWC, the stomata were almost completely closed, which severely affected vine transpiration. Determination coefficients (R^2^) of the relationships between physiological indicators and SWC were higher for gsw, followed by ψ_midday_ and A, equal to 0.84, 0.73 and 0.64, respectively. The intersection of the mean value of SWC calculated for the control vines with the function trajectory resulted in a reference value of -0.67 MPa, 0.17 mol m^-2^ s^-1^ and 16.0 µmol m^-2^ s^-1^ for ψ_midday_, gsw and A, respectively (**Figure 6**).

**Figure 7:**
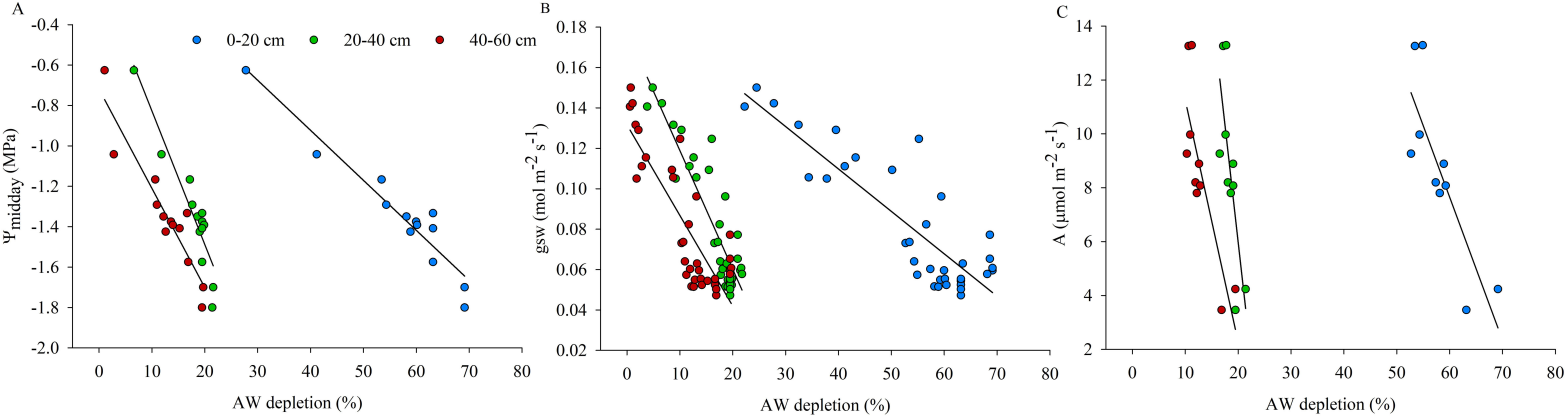
Distribution of midday stem water potential (ψ_midday_) **(A)**, stomatal conductance (gsw) **(B)** and photosynthesis (A) **(C)** of water-stressed (WS) vines in relation to available water (AW) depletion (%) considered in the 0-20, 20-40 and 40-60 cm soil layers during the experimental trial. Lines are only illustrative.

The analysis of plant measurements, carried out in the WS vines during the days of experiment of restricted irrigation, in relation to the progressive AW depletion reached in different soil layers (0-20, 20-40 and 40-60 cm) is shown in **Figure 7**. ψ_midday_ and gsw showed to be greatly affected by the dynamic of AW depletion according to soil depth, in particular, changes in plant water status and stomatal behavior are associated with higher reductions of the available water contained in the 0-20 cm soil layer, followed by the 20-40 cm and 40-60 cm soil layers (**Figures 7A, B**).

### 3.3 Water uptake by roots

**Figure 8:**
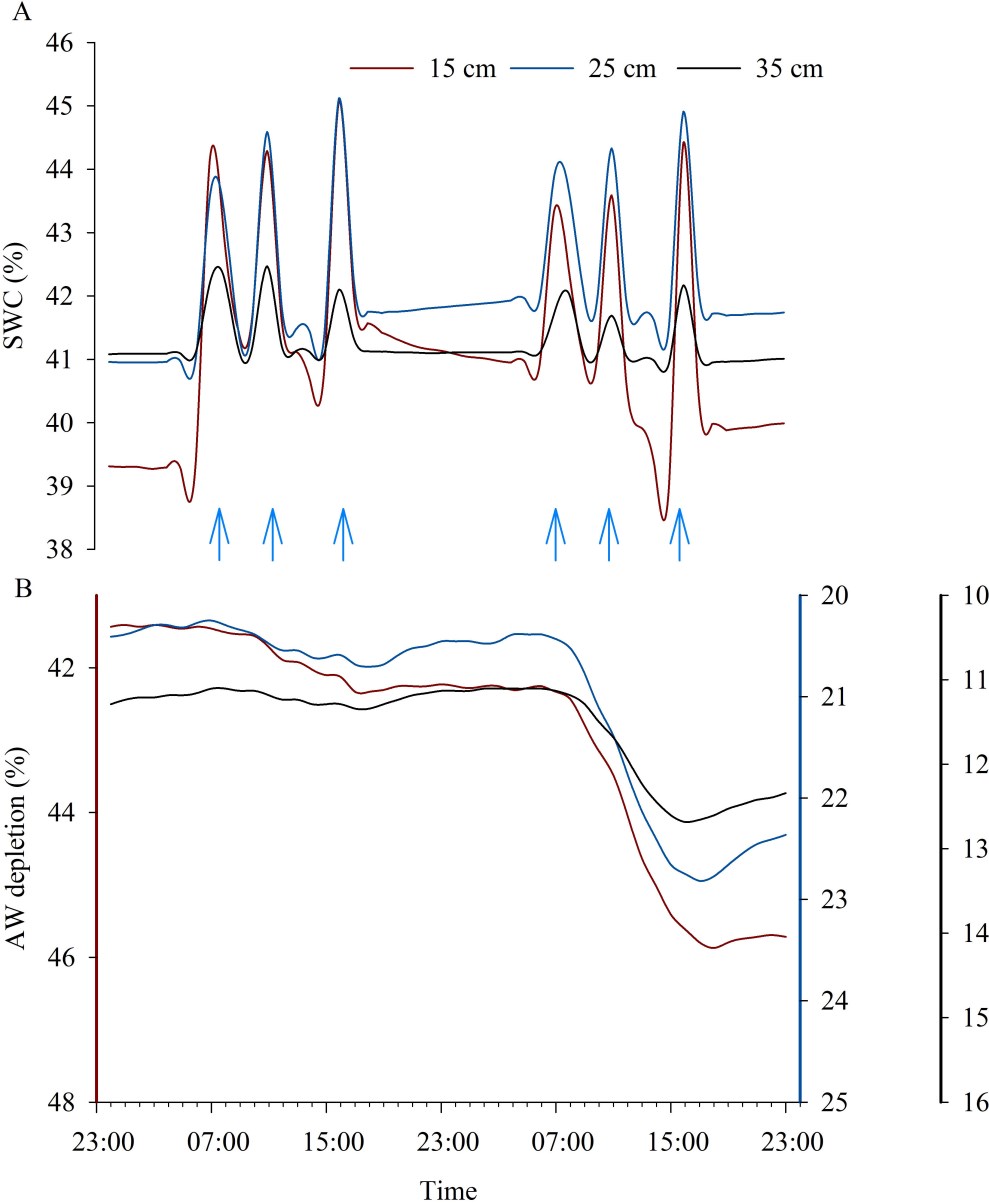
Daily courses of volumetric soil water content (SWC) in control vines **(A)** and of available water (AW) depletion in water-stressed vines **(B)** at 15, 25 and 35 cm soil depths on two consecutive representative days during the experiment (DOE 12 and DOE 13). Blue arrows indicate daily irrigation interventions carried out.

Daily SWC and AW depletion trends in C and WS vines, respectively, are shown in **Figure 8**. The changing pattern of SWC around the C vines reflected the combined effects of multiple daily irrigation interventions and the root water uptake strategy used by the vine. Greater variations in SWC were observed at 15, 25 and 35 cm soil depths (**Figure 8A**), while at deeper depths an almost flat trend in SWC was observed (data not shown). Three daily irrigation interventions were carried out to allow the vine to meet more efficiently the transpiration demand, which was higher during the midday hours, as shown by the increased root water uptake in the first 15 cm of soil on both days, but particularly on DOE 13, probably due to the increased environmental evaporative demand (**Figures 8A**, **1**).

The progressive decrease in SWC around the WS vines reflected a differential reduction in AW according to different soil depths and time of the day. Water uptakes were mostly concentrated in the central hours of the day (i.e., from 6:00 to 18:00), as shown by the decrease in AW contents when the vine accessed water to meet transpiration demands (**Figure 8B**). Preferential root water uptakes occurred in the superficial soil layers, as significant reductions of approx. 3.6%, 2.4% and 1.5% were achieved at 15, 25 and 35 cm soil depths during DOE 13, indicating a decrease in AW depletion with increasing soil depth (**Figure 8B**). Water uptake in the deeper layers increased gradually and slowly as the AW in the superficial soil layers was depleted.

**Figure 9:**
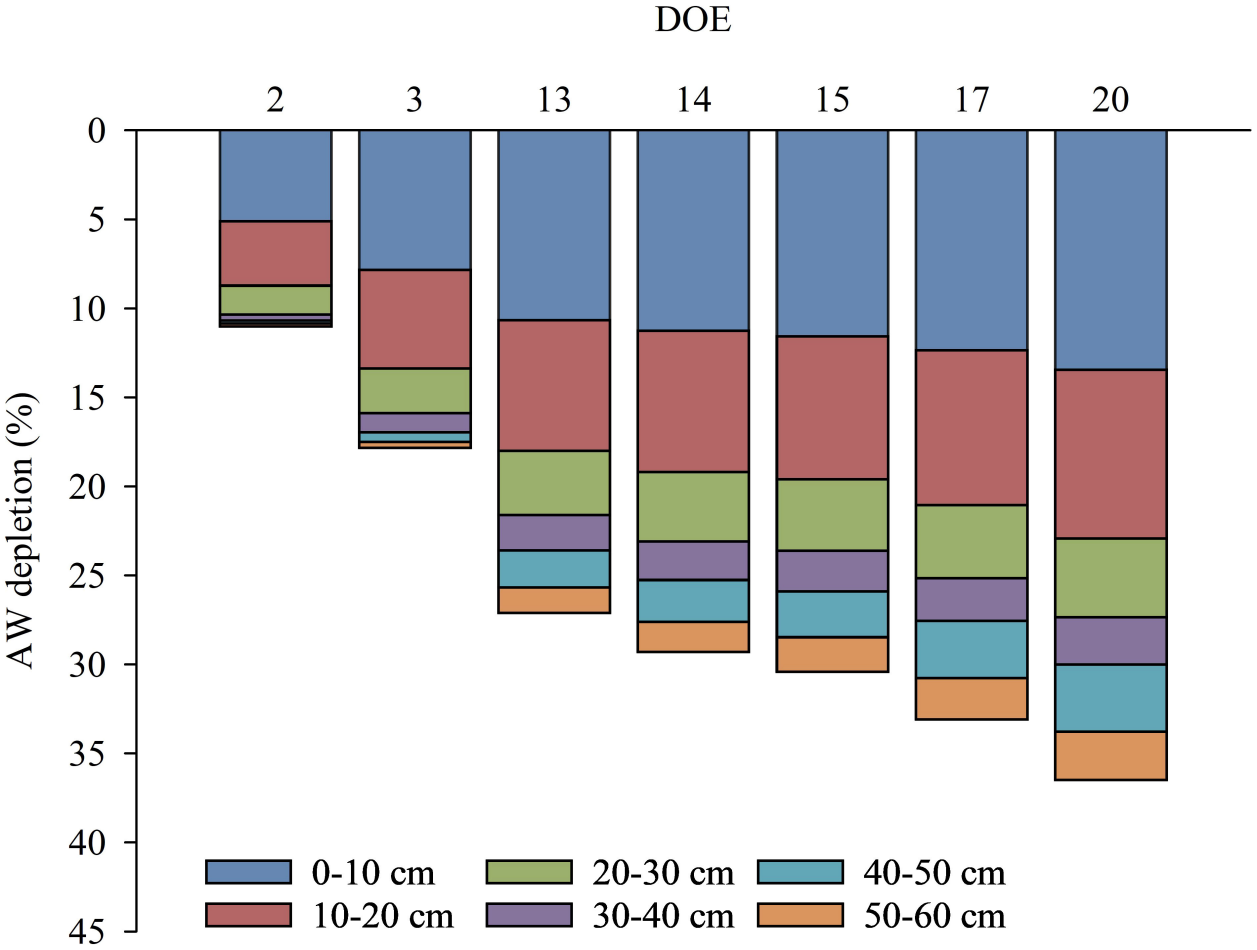
Dynamics of available water (AW) depletion in the whole soil profile (0-60 cm) reported for each 10 cm soil layer (0-10, 10-20, 20-30, 30-40, 40-50, 50-60 cm) during some representative days of the experiment (DOE).

The spatio-temporal changing dynamics of AW and the contribution of each layer to the progressive lowering of AW in the whole soil profile during the days of the experiment is shown in **Figure 9**. The AW was preferentially depleted in the first soil layers (up to 30 cm), which showed the largest reductions, contributing to a reduction in AW contained in the whole soil profile ranging from 10% to 27% during the days of the experiment. The progressive reduction of AW in the first soil layers (up to 30 cm) was slightly more limited from DOE 13 when the vines increased their water uptake in the deeper layers (from 30 to 60 cm), contributing to an AW reduction in the whole soil profile in the range of 0% to 9% during the days of the experiment (**Figure 9**). In particular, the deeper layers showed no or small reductions in AW at DOE 2 and DOE 3, respectively. From DOE 13, soil water conditions also began to be limiting in the deeper layers (from 30 to 60 cm), as the vines gradually accessed AW after the superficial soil layers had exhausted the more freely available water.

**Figure 10:**
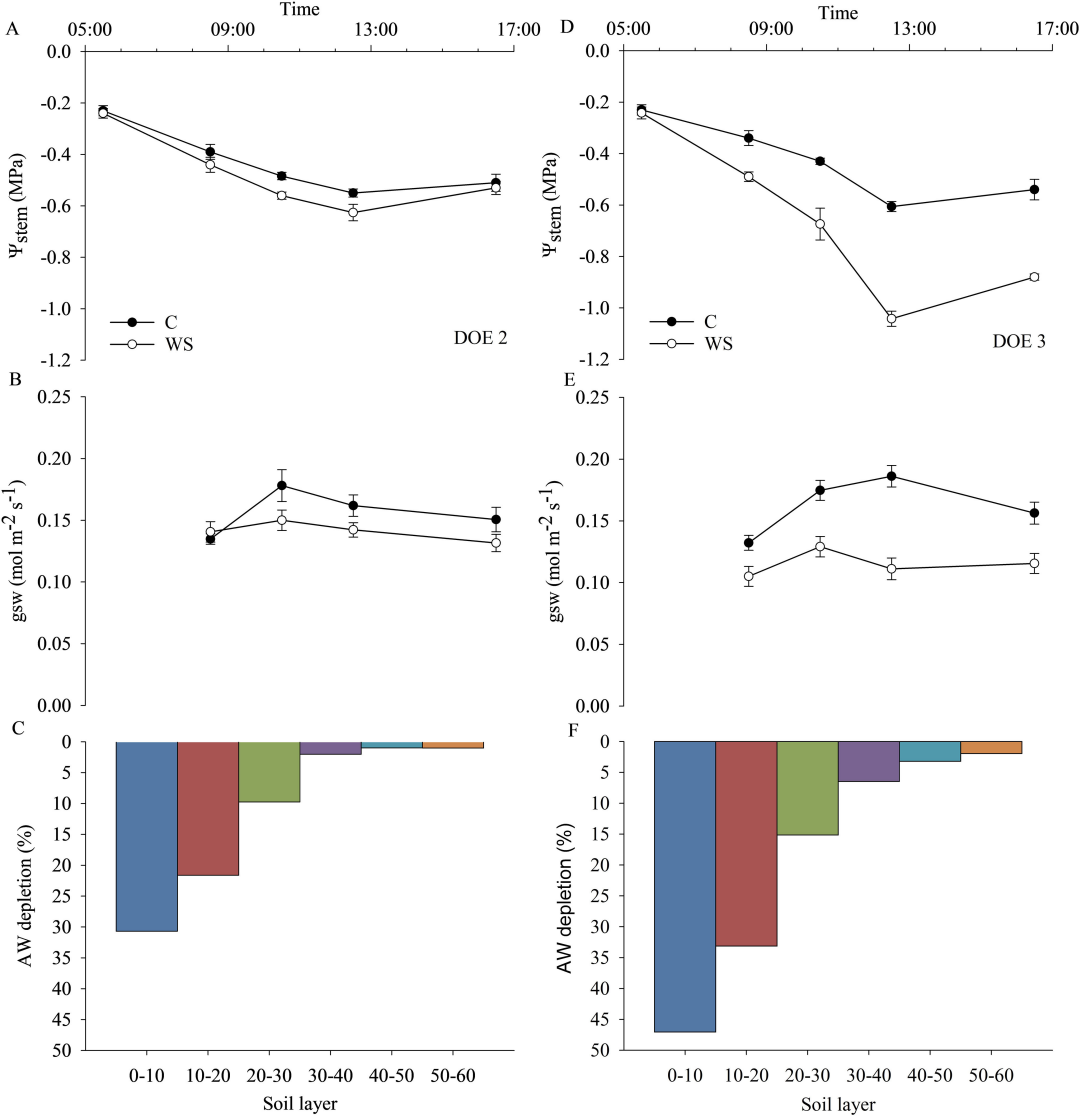
Daily dynamics of stem water potential (ψ_stem_), stomatal conductance (gsw) and available water (AW) depletion calculated in relation to the content of each 10 cm soil layer (0-10, 10-20, 20-30, 30-40, 40-50 cm) during the day before (DOE 2) **(A–C)** and at the onset of water stress (DOE 3) **(D–F)**.

Measurements of plant water status and stomatal conductance combined with soil observations and root water uptake dynamics during DOE 2 and DOE 3 are shown in **Figure 10**. Based on the daily dynamics of ψ_stem_ and gsw, DOE 2 was identified as the day before the onset of the water stress condition, since these parameters were almost the same in both C and WS vines (**Figures 10A, B**). During DOE 2, AW started to decrease in the first soil layers (up to 30 cm), with approximately 31%, 22% and 10% of AW being depleted in the 0-10, 10-20 and 20-30 cm soil layers respectively, while deeper layers showed insignificant decreases in AW (**Figure 10C**). Similarly, DOE 3 was identified as the day of water stress onset, as both gsw and ψ_stem_ of WS vines were different from those of C vines (**Figures 10D, E**). In particular, ψ_stem_ of the WS vines was equal to that of the C vines during the early morning hours, while it decreased significantly at midday, reaching a value of approx. -1.0 MPa, due to the combination of a higher evaporative demand and a rapid decrease in SWC, suggesting that the reduced water availability exacerbated the midday depression phenomenon (**Figures 10D, E**). During DOE 3, AW was further reduced by approx. 16%, 11% and 5% compared to DOE 2 in the 0-10, 10-20 and 20-30 cm soil layers, respectively (**Figure 10F**).

## 4 Discussion

### 4.1 Plant behavior under non-limiting soil water conditions

The irrigation strategy adopted for the well-watered vines consisted in splitting the daily irrigation volume into several interventions, maintaining the SWC steadily close to the FC (**Figure 8A**). The timely synchronization of the vine's water needs during the day, as influenced by environmental demands, with the irrigation supply, made it possible to guarantee an optimal soil water status at any time of the day, ensuring maximum root water uptake, avoiding the restriction of leaf gas exchange and the establishment of stomatal control and promoting transpiration processes (**Figures 3B, C**). This result is in agreement with Torres-Ruiz et al. (2016), who found that providing water at times when environmental conditions are more stressful, i.e. during the hottest hours of the day, allows kiwifruit vines to benefit from an improved water status and higher leaf gas exchanges at this time of the day. Soil water status and plant transpiration rate, which is influenced by environmental evaporative demand, are two main factors influencing plant water status at a given time of the day (Tardieu and Simonneau, 1998) (**Figures 3**, **4**, **7**). Stem water potential measured in the present study was used as the primary indicator of plant water status because it has been shown to be less variable than leaf water potential and more efficient in detecting small differences between irrigation treatments (McCutchan and Shackel, 1992). A gradient exists between leaves and stem that is proportional to the transpiration rate, resulting in lower leaf water potentials than stem water potentials, as previously reported in kiwifruit by Morandi et al. (2010). Therefore, when interpreting the data, it should be taken into account whether the results refer to leaf or stem water potential values. Maximum values of ψ_stem_ were observed at pre-dawn, when an equilibrium between plant and soil water potentials is achieved in the absence of water flux (Tardieu and Simonneau, 1998). ψ_stem_ reached lower values as the environment became more water-demanding (**Figure 4**), but well-irrigated vines maintained their ψ_midday_ in the range between -0.4 and -0.7 MPa, below which vines began to experience a stress condition that significantly altered normal plant functioning. Previous studies reported daily trends in leaf water potential for well-irrigated 'Hayward' kiwifruit ranging from -0.1/-0.2 MPa at pre-dawn to -0.9/-1.2 MPa at midday (Gucci et al., 1997), -0.03/-0.08 MPa at pre-dawn to -0.4/-0.8 MPa at midday (Judd et al., 1989) and -0.6/-1.0 MPa at midday (Montanaro et al., 2009; Calderón-Orellana et al., 2021), with differences depending on environmental evaporative demand (i. e. VPD conditions), stomatal behavior and root system development.

#### 4.1.1 Plant response to environmental demand

The plant functioning mechanism of the yellow-fleshed kiwifruit cultivar was first investigated by considering the relationships between ψ_stem_, gsw and E with the variable climatic conditions expressed by VPD, with soil moisture as a non-limiting factor. The increasing trend in VPD associated with climate change is recognized as one of the main climatic drivers affecting plant physiology (Grossiord et al., 2020). Kiwifruit vines showed diurnal fluctuations in ψ_stem_ in response to both evaporative demand (i.e. VPD) (**Figure 3A**) and light-induced stomatal opening (**Figure 4**) under non-limiting soil water conditions. Well-watered kiwifruit vines exhibited slight fluctuations in ψ_stem_ during the day (**Figure 4**), which contribute to the low hydrostatic pressure gradient between leaves and roots in kiwifruit (Dichio et al., 2013), indicating low hydraulic resistances in the water-conducting pathway (McAneney and Judd, 1983). ψ_stem_ declined with increasingly warm and dry environmental conditions under non-limiting soil water conditions (**Figure 3A**), but it is expected to become less responsive to changes in VPD as soil water availability begins to decrease. Vine transpiration was positively correlated with VPD (**Figure 3B**), as previously observed (Montanaro et al., 2007), and increased with environmental demands (Grossiord et al., 2020), thus slightly reducing potentials to the minimum values allowed, generally recorded during the hottest hours of the day, without affecting physiological performances (**Figure 4**). The gsw of well-watered vines, unlike ψ_stem_ and E, was not affected by the fluctuations in air VPD recorded during the days of the experiment, suggesting a plant behavior more likely to leave stomata open and allow high transpiration rates, which increased linearly with VPD, consequently lowering potentials (**Figure 3C**). A "midday depression" of gsw has been commonly reported in field-grown plants and attributed to high air VPD conditions (Tardieu and Simonneau, 1998). However, gsw reductions were generally not observed in well-watered plants, which showed almost constant gsw values despite high VPD, as previously described in kiwifruit (Gucci et al., 1996). This result is in agreement with Bardi et al. (2022), who studied the relationship between gsw and VPD in kiwifruit orchards (cv 'Hayward'), confirming a very low stomatal sensitivity of kiwifruit to VPD conditions and thus a tendency to anisohydric behavior. Stomatal sensitivity to air VPD affects the capacity of plants to regulate gas exchange in response to increasing VPD by controlling stomatal aperture as a strategy to decouple the canopy from the water-demanding atmosphere (Zweifel et al., 2007), and is highly variable between and within species (Grossiord et al., 2020).

### 4.2 Plant behavior under limiting soil water conditions

The response of yellow-fleshed kiwifruit to progressive SWC lowering was investigated to evaluate the effects of SWC on plant water status and physiological parameters and identify thresholds below which plant performance began to be compromised, up to severe effects.

#### 4.2.1 Plant water status

Vines exposed to a progressive reduction in soil water availability had decreasing ψ_stem_ values, revealing particularly severe stress conditions when significant differences from before dawn were observed. ψ_predawn_ is usually used as a reliable indicator of plant water status (Judd et al., 1989), which is influenced by the maximum soil water potential to which roots are exposed. The progressive decrease in ψ_predawn_ of WS vines indicated the inability of the plants to restore, during the nighttime hours, the tissue water reserves partially ceded to the transpiration flow during the day due to soil water shortage. The main physiological mechanisms of response to soil water deficit were reviewed, confirming that ψ_predawn_ decreases in response to increasing soil water deficit until the maximum level of water stress, identified as the time before irreversible damage and wilting of leaves occur, which was previously reported to range between pre-dawn leaf water potential values of -0.78 MPa and -1.06 MPa for green-fleshed kiwifruit (Gucci et al., 1997). A pre-dawn leaf water potential value of -1.0 MPa has been identified as the threshold below which vines are defined as 'severely stressed', with midday water potential values falling dramatically below those measured in well-watered vines (Judd et al., 1989; Gucci et al., 1997; Montanaro et al., 2007), while a value of -0.3 MPa has been identified as the threshold below which gsw begins to be affected (Gucci et al., 1996). Although plants subjected to decreasing soil water availability showed reduced ψ_predawn_, which can be used as a reliable indicator of the maximum soil water potential to which the roots are exposed (Tardieu and Simonneau, 1998), ψ_predawn_ values are not always sufficient to detect water stress in kiwifruit, as differences in gsw can be found even when differences in water potentials are not appreciable. This was also evident in the current study, particularly on DOE 3, where ψ_stem_ values were the same between C and WS vines at predawn, but differed significantly at midday (**Figures 2**, **10D**), reflecting a condition of early mild stress, also supported by a reduction in gsw of approx. 40% at midday (**Figure 10E**). Therefore, ψ_midday_ and gsw could be considered as more sensitive indicators of early stages of water stress in yellow-fleshed kiwifruit. This is in agreement with Gucci et al. (1996), who found that ψ_predawn_ was insensitive to the initial phase of soil water content decrease and was significantly affected only after reaching the threshold value of -0.3 MPa.

#### 4.2.2 Stomatal activity

Decreasing soil moisture induces plants to regulate stomatal conductance, in order to avoid damage to the hydraulic system, and the intensity of gsw reduction depends on plant stomatal behavior, ranging from anisohydric to isohydric strategies (Tardieu and Simonneau, 1998; Martínez-Vilalta et al., 2014; Grossiord et al., 2020). Whether kiwifruit vines have an isohydric or anisohydric behavior is still highly discussed, but it is increasingly accepted that isohydricity is cultivar rather than species specific, and is also influenced by external conditions and the peculiar environments in which plants grow. Previous studies have found that gsw of 'Hayward' kiwifruit is reduced by stomatal closure under severe water stress (Chartzoulakis et al., 1993; Gucci et al., 1996). Gucci et al. (1997) found smaller differences between leaf water potentials of stressed and well-irrigated vines when measured at midday than at pre-dawn, supporting the conclusion that 'Hayward' kiwifruit responds conservatively to soil moisture depletion by closing stomata, limiting transpiration and thus the decline in leaf water potential at midday (tendency to isohydric behavior), which is not in agreement with other research that suggest a lack of or poor stomatal control of transpiration, with leaves continuing to lose water as soil water deficit increases (Judd et al., 1989; Calderón-Orellana et al., 2021). In particular, Montanaro et al. (2007) suggested that 'Hayward' kiwifruit can cope with the Mediterranean environment by adopting an adaptive strategy aimed at low gsw and carbon gain sacrifice for water conservation and photoprotection, also under severe water stress, disapproving the absence of stomatal control. From the measurements carried out in the present study, the differences between ψ_stem_ of C and WS vines were greater at midday (**Figure 4**), suggesting that yellow-fleshed kiwifruit tends not to have the same stomatal behavior as that found by Gucci et al. (1997) in green kiwifruit, still slightly increasing E during the hottest hours of the day and significantly decreasing potentials, although there was a consistent reduction in gsw of WS vines, which was only 25-44% of that of C vines throughout the experimental trial (**Figure 4**), in agreement with Gucci et al. (1997). Therefore, the gsw of yellow-fleshed kiwifruit vines appeared to be strongly affected by changes in soil water status (**Figure 6B**), in agreement with Gucci et al. (1996), identifying SWC as a key driving variable involved in the behavior of kiwifruit vine stomata. Stomatal behavior of kiwifruit has been reported to be highly sensitive to soil and air hydration, contributing to minimize leaf water deficits and regulate overall canopy transpiration (Gucci et al., 1996). An average value of gsw reduced by 65% compared to C vines was achieved when ψ_midday_ was lower than -0.8 MPa (**Figure 5A**), confirming the high stomatal sensitivity to changes in plant water status, as previously reported by Gucci et al. (1996). The relationship between gsw and ψ_midday_ suggests that the stomatal response occurs after a threshold value of plant water potential is reached (Garnier and Berger, 1987), showing a progressive closure of stomata when ψ_stem_ falls below the critical value (**Figure 5A**). This result, obtained under field conditions, is consistent with the finding of Mills et al. (2009), who investigated the water stress physiology of pot-grown yellow-fleshed kiwifruit and identified a midday leaf water potential value of approx. -1.0 MPa as the threshold below which gsw began to be significantly affected (reduced by approx. 80% compared to control vines when leaf water potential reached values of -1.5 MPa). At the same time, it does not fully agree with Boini et al. (2022), who identified -0.5 MPa as the midday stem water potential value of field-grown vines below which *A. chinensis* var. *chinensis* reduces all leaf gas exchange parameters. Several research studies agreed that A and gsw of field-grown vines follow trends parallel to those of container-grown vines, but the stress develops more gradually when irrigation is restricted in the field (Gucci et al., 1996), also due to the generally lower unsaturated hydraulic conductivity of pot substrates compared to natural fields (Tardieu and Simonneau, 1998). It is expected that under field conditions, the severity of water stress can be mitigated and vines perform better due to the more extensive and developed root system, which explores a larger soil volume and compensates for the effects of water stress by absorbing water from greater soil depths (Goldhamer et al., 1999; Mills et al., 2009).

#### 4.2.3 Sensitivity to drought stress and stem water potential threshold

The diurnal changes observed in leaf E reflected the changes in gsw, suggesting that stomatal closure was an adaptive strategy to prevent leaf dehydration by slowing transpiration (**Figure 4**). Despite the relatively early adaptive response of stomata, which limits further decreases in water potential and ensures that leaf water demand does not exceed the supply capacity of the hydraulic system (Martin-StPaul et al., 2017), ψ_stem_ was not protected and decreased significantly, probably due to the increased hydraulic resistance of progressively drying roots as reported by Gucci et al. (1996). The reduction in ψ_stem_ was related to the progressive AW depletion (**Figure 7A**) and could therefore be dependent on the increased root hydraulic resistance. Soil drying has been reported to induce changes in root hydraulic conductance, which decreased rapidly but reversibly upon re-watering (Vandeleur et al., 2009; Black et al., 2012). In well-watered kiwifruit vines T_leaf_ was higher than air temperature, with large differences indicating a reduced capacity for evaporative water loss as previously reported in 'Hayward' kiwifruit (Buwalda et al., 1992; Montanaro et al., 2007). However, ΔT decreased with increasing environmental demand (i.e. VPD) due to higher transpiration rates, resulting in increased evaporative water loss and cooling effect observed in kiwifruit vines under non-limiting soil water conditions (**Figure 3D**). Reduced transpiration induced by water shortage compromised the thermoregulatory capacity of leaves, leading to a further increase in leaf temperature, approx. 2 to 4°C higher than kiwifruit vines under non-limiting soil water conditions (**Figure 5B**). In kiwifruit, the small gradient between canopy and soil water potentials probably decreases water uptake, effectively reducing the ability of this species to tolerate drought (Gucci et al., 1996). Hydraulic conductivities measured for both root and stem xylem tissues are higher compared to other species, and the resistance to water movement within the vine is very low (McAneney and Judd, 1983; Dichio et al., 2013). This supports that the soil to root pathway is the main resistance to water uptake by the kiwifruit root system, even at high soil water potentials, which can be worsened by soil drying (McAneney and Judd, 1983). Although high whole-plant hydraulic conductance in kiwifruit contributes to high transpiration rate and photosynthetic capacity, it can also lead to increased susceptibility to some drought-related hydraulic impairments (e.g. cavitation, embolism), which are exacerbated during drought and in Mediterranean environments (Dichio et al., 2013; Bardi et al., 2022). Most of the studies on kiwifruit water relations and needs refer to *A. chinensis* var. *deliciosa*, while information on the physiology of yellow-fleshed kiwifruit under non-limiting soil water conditions and reduced soil water availability is emerging recently. The results obtained in the present work show a change in the behavior of kiwifruit vines exposed to different soil water conditions. In particular, kiwifruit vines moved from a high water-consuming approach, observed under non-limiting soil water conditions, without limiting their activities or suffering from critical environmental conditions (e.g. high VPD), to a more conservative adaptive strategy adopted under conditions of limited soil water availability. The current study suggests the identification of a threshold value of the plant water status, influenced by different soil water conditions, which can be considered critical for *A. chinensis* var. *chinensis*, i.e. ψ_midday_ values below -0.8 MPa indicated an initial impairment of leaf gas exchange activity (**Figure 5A**). From the measurements carried out in the present study, the ψ_stem_ variations of WS vines from the control values during DOE 3, reaching a value of -1.0 MPa at midday, identify the establishment of a stress condition. Instead, ψ_predawn_ and ψ_midday_ values of -1.0 and -1.8 MPa, respectively, corresponding to the maximum level of water stress achieved during the experimental trial, already identify a severe water stress condition in yellow-fleshed kiwifruit. Threshold values reported for *A. chinensis* var. *chinensis*, below which physiology appeared to be compromised, were higher than those reported for *A. chinensis* var. *deliciosa* in a typical Mediterranean environment (Montanaro et al., 2009), supporting the lower tolerance of yellow-fleshed kiwifruit to reduced water availability compared to green-fleshed kiwifruit, cv 'Hayward'. This is consistent with the findings of Rahman et al. (2011), who demonstrated that *A. chinensis* var. *deliciosa* had a higher water loss than *A. chinensis* var. *chinensis* during a mild or short-term period of water stress, but a higher tolerance and then greater advantages under conditions of severe or prolonged water stress.

### 4.3 Spatio-temporal dynamics of soil moisture and thresholds for precision irrigation

Monitoring soil water status, which is related to plant water status, is increasingly becoming a common approach to schedule and control irrigation in order to ensure optimal plant performances (Nolz et al., 2016). Fluctuations in soil AW vary throughout the soil profile, depending on the structure and development of the root system, the irrigation system wetting a specific soil volume, and the environmental conditions (such as reference evapotranspiration and VPD). Therefore, it is important to define *in-situ* soil moisture thresholds based on multi-layer sensor data collected in the field to manage irrigation from a practical point of view. The method proposed in this paper defines thresholds for AW by measuring plant responses to progressive lowering of SWC and analyzing their relationships, in order to identify the 'stress point' that serves to early detect a water stress condition and implement more sustainable and precise irrigation strategies. The present study found that plant water status and other physiological indicators of kiwifruit vines were sensitive to a progressive decrease in SWC (**Figure 6**). This result is consistent with the findings of previous research carried out on other species (Garnier and Berger, 1987; Naor and Cohen, 2003; Al-Yahyai, 2012; Jamshidi et al., 2020), focusing on the response of water stress indicators to soil drying and whether these parameters are correlated with each other. The resulting correlation confirmed that, in addition to environmental variables, other factors such as soil moisture could have a great influence on plant water status. In the lower range of SWC, plant water status and physiological parameters were controlled by the root water uptake mechanism and water availability. The current work aimed to identify soil water content thresholds and define the amount of AW that can be used by the plant without compromising the physiological aspects, delimiting the range where no stress should occur. Results provide new information to define the depletion fraction of AW from the root zone before moisture stress occurs in yellow-fleshed kiwifruit, which was previously considered to be 0.35 for kiwifruit (cv 'Hayward') with a crop evapotranspiration (ET_c_) of approx. 5 mm day^-1^ (Allen et al., 1998). Vines, which absorbed water differentially in each soil layer, experienced a decrease in the AW amount of approx. 35 m^3^ ha^-1^ from the soil profile achieved at DOE 2, identified as the day until which the plant's water status and physiology were maintained at optimal levels, which corresponds to a depletion of about 11% of the AW contained in the whole soil profile (0-60 cm) (**Figure 9**). Instead, AW was overall reduced by approx. 17.8% at DOE 3, identified as the 'stress onset day' (**Figures 10D, E**), which implied a decrease of about 57 m^3^ ha^-1^ of AW from the whole soil profile (**Figure 9**). The depletion factor changes with the environmental evaporative demand, in particular at high ET_c_ it is 10-25% lower than the ordinary value, indicating that a lower AW fraction can be depleted before the onset of the stress (Allen et al., 1998). Values found in the present study highlighted that under the same ET_c_ conditions, the depletion factor of AW for yellow-fleshed kiwifruit was approx. 50% lower than that reported for 'Hayward' in the FAO-56 paper. A previous study has focused on defining the allowable soil water depletion for precise irrigation scheduling in other tree crops, and obtained a depletion factor lower than those tabulated in the FAO-56 paper (Puig-Sirera et al., 2021). The assessment of root water uptake through changes in soil moisture is promising (Jackisch et al., 2020) and provides further information on the amount of water depleted, which can be considered complementary in estimating transpiration and water needs of plants. The greater activity of surface roots is shown by the rapidly changing pattern of both SWC around the C vines and AW depletion around the WS vines, which followed a decreasing trend with increasing soil depth (**Figure 8**). This is consistent with previous research investigating the pattern of SWC at different depths in the root zone of kiwifruit vines, which showed that vines preferentially use near-surface water when available (Clothier and Green, 1994; Green and Clothier, 1995; Green et al., 2006). Preferential root water uptake occurred initially from the superficial, wetter parts of the soil, and then increased in the deeper layers. Vines began to extract water from the depths where water was more freely available (**Figure 9**), in accordance with Green and Clothier (1995) who confirmed the capacity of kiwifruit vines to shift their water uptake pattern in response to changes in soil water availability. In the present study, soil water depletion was used to understand the intensity and dynamics of root water uptake throughout the root zone. Considering the dynamics of root water uptake, soil moisture thresholds should be identified for each layer, which contribute differently to triggering water stress. Therefore, in Mediterranean environments where daily vine water requirement can reach about 60-70 m^3^ ha^-1^ during the hottest days, it is advisable to split the daily irrigation volume in two or more interventions, according to the DOE 2 depletion indication. Ensuring RAW throughout the day in the first 0-30 cm soil layer, mainly interested by root water uptake, is essential to avoid the establishment of water stress and in particular of midday depression phenomena.

## 5 Conclusions

The analysis of stem water potential and leaf gas exchange of yellow-fleshed kiwifruit showed that the atmospheric condition (VPD) did not affect stomatal closure/opening under non-limiting soil water conditions, indicating that vines do not implement stomatal control preferring to transpire, losing water according to its availability and thus lowering the potentials. Yellow-fleshed kiwifruit is then characterized by a high water-consuming behavior when soil water availability is not limiting, outlined in a Mediterranean environment. However, stomata appeared to be sensitive to changes in soil water status, decreasing their conductance as soil water content progressively declined, representing a protective and adaptive strategy to water deficit. The approach based on both soil water monitoring and determination of plant physiological parameters was used to identify soil moisture thresholds of high practical applicability for irrigation management. Monitoring of stem water potential and stomatal activity throughout the experiment made it possible to identify the point before and at the onset of water stress, below which plant water status and physiological activity began to be affected, and to define *in situ* irrigation thresholds. The results of the study suggest that the AW that can be depleted before water stress occurs is approximately 11% of the AW of the 0-60 cm soil layer, which appears to be lower than the standard value reported in the FAO-56 paper. The spatio-temporal dynamics of root water uptake highlighted a different contribution of each soil layer to meeting the transpiration demands of the vines. The decrease in AW was more pronounced in the superficial soil layers (i.e. 0-30 cm), which are mainly affected by root water uptake, contribute more to the onset of stress and should therefore be carefully managed by irrigation. Although the experimental trial was carried out in a semi-arid Mediterranean environment, the approach can be transferred to other environments. Future research is needed to define and validate information on the stress coefficient (Ks) and the AW depletion fraction throughout the irrigation season and in different soil and environmental conditions, in order to develop precision irrigation strategies that allow plants to meet transpiration demand and prevent the establishment of temporary water stress conditions, which are particularly common in Mediterranean-type climates.

## Data Availability

The raw data supporting the conclusions of this article will be made available by the authors, without undue reservation.
